# Phenomenological insights into the FLASH radiotherapy-induced abscopal effect

**DOI:** 10.3389/fonc.2025.1657392

**Published:** 2025-09-17

**Authors:** Paolo Castorina, Gianluca Ferini, Francesco Romano

**Affiliations:** ^1^ Istituto Oncologico del Mediterraneo, Viagrande, Italy; ^2^ Istituto Nazionale Fisica Nucleare (INFN), Sezione di Catania, Catania, Italy; ^3^ Faculty of Mathematics and Physics, Charles University, Prague, Czechia; ^4^ REM Radioterapia, Viagrande, Italy; ^5^ Department of Medicine and Surgery, University of Enna Kore, Enna, Italy

**Keywords:** mathematical modeling, Gompertz law, radiotherapy, abscopal effect, FLASH radio-therapy immunotherapy

## Abstract

**Background:**

The abscopal effect suggests that the impact of radiotherapy extends beyond the direct tumor local regression, due to activation of the immune response. Its effectiveness may vary depending on whether high- or low-radiation doses are used. In FLASH therapy, the high-dose rate treatment induces systemic effects that may trigger an abscopal response.

**Methods:**

We discuss a phenomenological, computational model, based on available *in vivo* FLASH radiotherapy data, to quantitatively analyze the possible synergistic effects with the immune system to produce a systemic effect.

**Results:**

The method enables a quantitative assessment of the interaction between FLASH radiotherapy and the activated immune response, based on observations of metastatic shrinkage due to the FLASH treatment of the primary tumor.

## 1 Introduction

Radiotherapy remains one of the most effective local treatments for cancer. In addition to directly inducing cell death, ionizing radiation is now recognized to play a complex modulatory role on the tumor microenvironment [for a recent comprehensive review, see ref (1)]. In particular, radiation-induced DNA damage and the subsequent release of damage-associated molecular patterns (DAMPs) activate proinflammatory pathways and recruit innate and adaptive immune effectors, including macrophages and cytotoxic T lymphocytes. This immunomodulatory effect may have a significant impact not only on the primary tumor but also on distant, non-irradiated lesions, a phenomenon known as the abscopal effect (2, 3).

Experimental and clinical observations have reported abscopal responses under particular conditions, including high-dose irradiation schemes (4–14). In this context, FLASH radiotherapy—a novel technique delivering ultra-high rates—has emerged as a promising modality capable of triggering such systemic responses while sparing normal tissues (15–19).

Notably, preclinical studies on mice have documented a biphasic response following a single FLASH dose of 25 Gy to the primary tumor, characterized by an initial regression, a transient regrowth, and a delayed second phase of tumor reduction (20). For a recent review on preclinical studies with FLASH therapy, see ref (21).

Understanding the mechanisms underlying this temporal pattern is essential to elucidate the potential of FLASH therapy as a systemic treatment. However, direct modeling of the full complexity of the biological cascades involved—including immune cell trafficking, antigen presentation, and tumor–immune interactions—remains a formidable challenge due to the limited availability of detailed *in vivo* data.

To address this, we adopt a phenomenological modeling strategy grounded in experimental observations. The present work introduces a computational framework that builds upon prior *in vivo* studies of FLASH therapy and aims to capture both local and systemic tumor responses. The approach relies on two key components: 1) a previously proposed parametrization of FLASH-induced effects based on tumor volume data (22) and 2) a dynamical model incorporating tumor growth and its modulation via radiation-activated immune response. Importantly, the model is formulated to account for both primary and metastatic tumor sites, with attention to the eventually delayed, systemic effects of localized irradiation. The method is completely general and can be applied to any tumor phenotype.

Tumor growth is modeled using the Gompertz law (23–26), which captures the decelerating expansion of tumors due to intrinsic regulatory mechanisms. Radiotherapy effects are introduced as a time-dependent term modifying the growth rate (22), calibrated against FLASH experimental data in which the dose is given in short pulses at ultra-high dose rate on lung fibrosis in mice, based on a linear electron accelerator (20). The phases observed after irradiation are hypothesized to result from immune activation, and their impact on distant metastases is incorporated through a coupling term modulated by parameters governing the strength and timing of the systemic immune response.

By comparing the predicted dynamics of tumor volumes at primary and metastatic sites, the model allows for a quantitative assessment of how FLASH-induced immune mechanisms may lead to the observed abscopal effects. Therefore, this study provides a theoretical framework to interpret experimental data and supports the hypothesis that FLASH therapy, under specific dosing conditions, can induce an effective systemic antitumor response.

More precisely, the progression of the metastasis depends on two independent parameters, which describe respectively the local initial conditions (of the distant site) and the initial size of the metastasis at the onset of the systemic immune effect, induced by the FLASH therapy applied to the primary tumor. A possible correlation between them implies a metastasis size dependence of the immune response. Moreover, a time delay in immune response between the primary tumor and its metastasis suggests a dynamics not reducible to a one-compartment pharmacokinetic model.

## 2 Methods

The proposed approach relies on two key components: 1) a previous analysis of *in vivo* data on FLASH therapy, which supports a phenomenological parametrization of its effects (20, 22), and 2) a computational framework that models the immune response activated by radiotherapy. See refs (27–30). for the mathematical model of the synergy between radiotherapy and the immune system.

### 2.1 Computational method

The untreated tumor growth is described by the Gompertz law (GL) (23–26), using the following equation [see Equation S2 in the **Supplementary Material**]:


(1) $$\frac{1}{N(t)}\frac{dN(t)}{dt}=a-k\ln\;\frac{N(t)}{N_{0}}=k\ln\;\frac{N_{\infty }}{N(t)}$$

where *N* is the cell number (proportional to the volume for a constant density solid tumor), and *a*, *k*, and *N*_0_ are constants, indicating the exponential growth, the feedback effect, and the initial cell number, respectively. *N*_∞_ is the carrying capacity (*N*_∞_ = *N (0*)exp(*a/k*)), i.e., the maximum number of cells according to the boundary conditions of the growth. After the seminal paper by L. Norton (24), the GL has been extensively applied to describe *in vivo* and *in vitro* data [see ref (26). for a recent review and ref (31). for a complete compilation for various phenotypes: bladder, breast, colon, lung, ovarian, pancreatic cancer, head and neck squamous cell carcinoma, hepatocellular carcinoma, melanoma, and renal cell carcinoma]. For untreated tumors, the GL emerges from microscopic, biological mechanisms where natural/adaptive immunity is taken into account (32, 33).

The radiotherapy effect can be incorporated directly into the previous equation by


(2) $$\frac{1}{N_{p}(t)}\frac{dN_{p}(t)}{dt}=k_{p}\ln\;\frac{N_{\infty }^{p}}{N_{p}(t)}-F(t)$$

where the index *p* = primary. The function *F*(*t*) contains all the direct and long-term effects of radiotherapy, i.e., the initial cell killing effect of radiation plus the triggered immune response.

In ref (22), the following parametrization of *F*(*t*), driven by the FLASH data of ref (20), has been proposed


(3) $$F(t)=c_{0}+c_{1}e^{-c_{2}t}-c_{f}t.$$

The four parameters depend on dose, *d*, as reported in Table 1 of ref (22). It is worth emphasizing that the data from ref (20), referring to a model of lung fibrosis in mice, are used as representative of the effects of FLASH therapy. The value of the parameters depends on the tumor phenotype and the microenvironment, but the chosen parametrization (22), based on the four parameters, is sufficiently flexible to account for the various local factors that influence tumor evolution after treatment.

**Figure 1:**
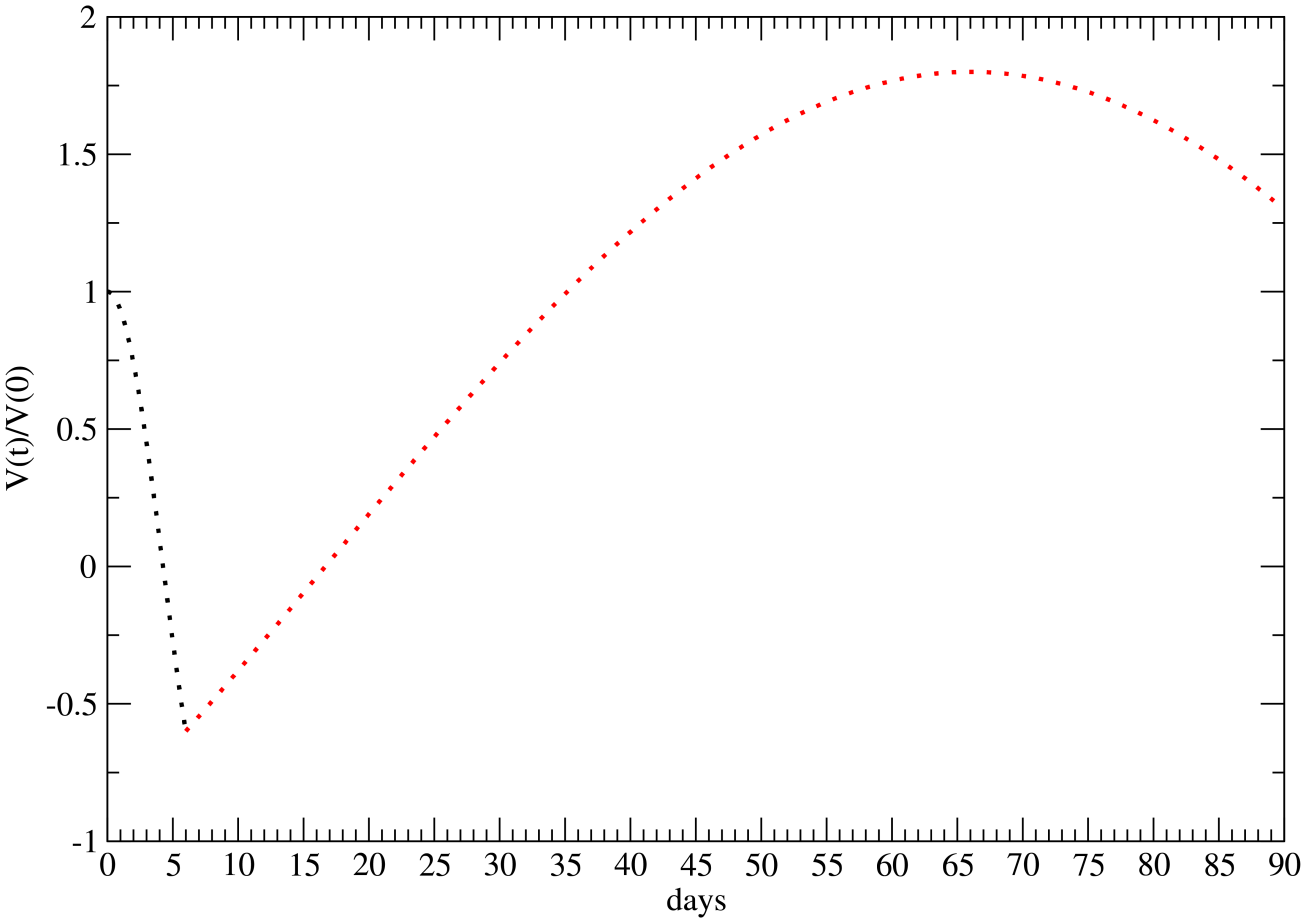
Qualitative behavior of the time evolution of the FLASH therapy effect for 25 Gy (22). Initial cell killing effect—black points; regrowth and late time regression—red points. The minimum and maximum are respectively at *t*_1_ = 5 days and *t*_2_ = 66 days after the administration.

In particular, a key feature of the experimental data (20) is that, for a high-dose rate (25 Gy), the tumor progresses toward complete recovery (CR). This behavior corresponds to a change in the sign of the parameter *c_f_*, which captures the late time effect. Specifically, the data in (20) reveal an initial phase of tumor cell killing, described essentially by *c*_0_ and *c*_1_ (22), followed by a period of regrowth, beginning at time *t* = *t*_1_, and a phase of tumor regression starting at *t* = *t*_2_, as qualitatively illustrated in **Figure 1**.

The function *F*(*t*) describes, macroscopically, the therapy-induced effects, which originate from the underlying microbiological pathways. However, due to the lack of knowledge of all the biological effects of the FLASH therapy, in the phenomenological approach (22), the initial drop in tumor volume is essentially attributed to the direct, immediate, radiation action, which decreases over time. The subsequent regrowth phase suggests that cellular repair mechanisms begin to prevail, counteracting the initial radiotherapeutic impact. The long-term behavior is mainly governed by the parameter *c_f_*. Therefore, after the initial phase, the therapeutic outcomes are related to other dynamic mechanisms induced by radiation, such as immune activation.

**Figure 2:**
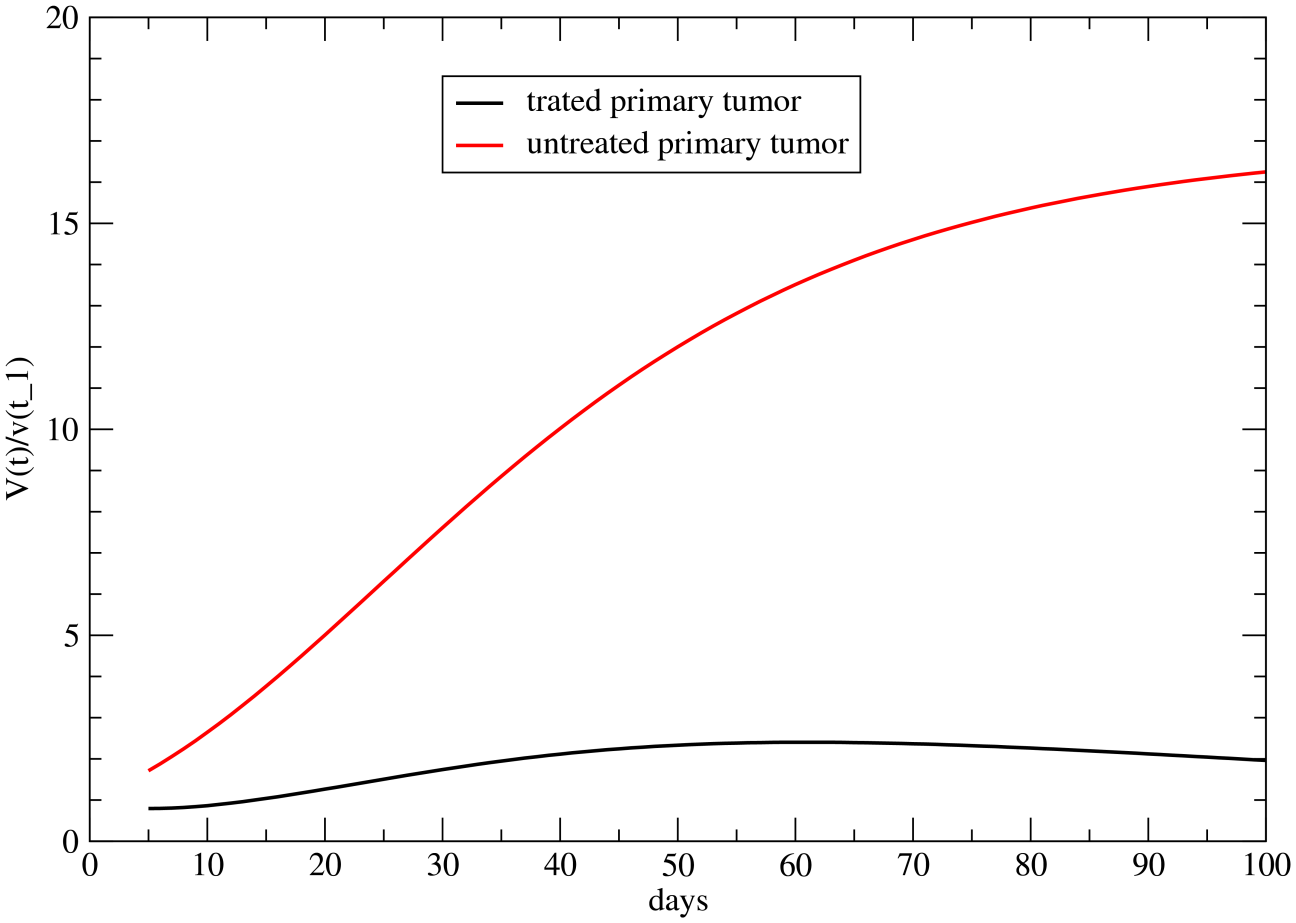
Volume variation for *t > t*_1_, for the untreated primary tumor versus the primary with FLASH therapy. Parametrization in ref (22).

In fact, the regrowth rate is significantly slower than that observed in untreated tumors (see **Figure 2**). Even more striking is the long interval between *t*_1_ and *t*_2_, with *t*_1_ occurring 5–6 days after treatment and *t*_2_ at 66 days. This slow regrowth and the delay of the primary tumor regression strongly support the hypothesis that the observed evolution/regression is driven by an immune response activated by FLASH radiotherapy, which not only protects normal tissues via immune modulation (34) but also enhances the infiltration of CD8^+^ T cells into the tumor and triggers a variety of immune responses in the spleen (35). These significant results support the potential of combining FLASH radiotherapy with immune checkpoint inhibitor (ICI) therapy (36).

Let us now consider a metastatic lesion located far from the primary tumor. Without direct immunotherapy, it is reasonable to assume that the metastatic growth rate for *t > t*_1_ is similar to that of the primary tumor, due to the systemic-induced immune response. However, the specific Gompertzian growth rate of the metastasis (M) depends on two parameters: *k_M_* and the carrying capacity $N_{\infty }^{M}$ . *k_M_* reflects the internal feedback mechanisms during its evolution and can be assumed equal to *k_p_*, the one for the primary tumor. Vice versa, the metastatic carrying capacity $N_{\infty }^{M}$ may vary due to local conditions, such as nutrient availability, tissue environment, and other site-specific factors.

Therefore, for the metastatic cells, *N^M^* (*t*), the evolution equation turns out to be (*k_p_* = *k_M_* = *k*)


(4) $$\frac{1}{N_{M}(t)}\frac{dN_{M}(t)}{dt}=k\ln\;\frac{N_{\infty }^{M}}{N_{M}(t)}-Y(t)F(t)$$

where *Y*(*t*)*F*(*t*) describes the immune response in the metastatic site activated by FLASH therapy on the primary tumor.

The initial cell killing effect is caused by radiation on the primary tumor and does not occur at the metastatic site. The subsequent regrowth, starting at time *t*_1_, indicates that the direct radiation effect has become negligible. Moreover, the regrowth rate remains significantly lower than in the untreated case, as shown in **Figure 2**. Assuming this slower progression is a systemic effect leads to a similarly reduced growth rate in the metastasis.

Nonetheless, as previously discussed, the carrying capacity and the initial cell number of the primary tumor can differ substantially from those of the metastatic site.

The quantitative difference can be better understood by comparing the GL for the primary volume *V_p_* (for a constant density tumor (see Equations 1–3)), given by, for *t > t*_1_ (see the **Supplementary Material**)


(5) $$\frac{V_{p}(t)}{V_{p}(t_{1})}=exp[(\ln\;\frac{V_{\infty }^{p}}{V^{p}(t_{1})}-c_{0}/k-c_{f}/k^{2})(1-e^{-k(t-t_{1})})-e^{-(c_{2}t_{1})}\frac{c_{1}}{k-c_{2}}(e^{-c_{2}(t-t_{1})}-e^{-k(t-t_{1})})+(t-t_{1}e^{-k(t-t_{1})})c_{f}/k]$$

with the analogous GL for the metastasis (see Equation 4),


(6) $$\frac{V^{M}(t)}{V^{M}(t_{1})}=exp[(\ln\;\frac{V_{\infty }^{M}}{V^{M}(t_{1})}(1-e^{-k(t-t_{1})})-\int_{t_{1}}^{t}dt^{\prime }Y(t^{\prime })F(t^{\prime })e^{-k(t-t^{\prime })}$$

Indeed, one can write


(7) $$\ln\;\frac{V_{\infty }^{M}}{V^{M}(t_{1})}=\ln\;\frac{V_{\infty }^{p}}{V^{p}(t_{1})}+\ln\;\lambda$$

where the parameter (see Equations 5, 8)


(8) $$\lambda =\frac{V_{\infty }^{M}}{V_{\infty }^{p}}\frac{V^{p}(t_{1})}{V^{M}(t_{1})},$$

is related to the metastasis size and can be >1 or <1. However, since, from the previous equation,


(9) $$\lambda (\frac{V_{\infty }^{p}}{V^{p}(t_{1})})=(\frac{V_{\infty }^{M}}{V^{M}(t_{1})}),$$

*λ <* 1 is a more realistic value.

By previous definitions, Equation 7. becomes


(10) $$V^{M}(t)/V^{M}(t_{1})=exp[(\ln\;\frac{V_{\infty }^{p}}{V^{p}(t_{1})}+\ln\;\lambda )(1-e^{-k(t-t_{1})})-\int_{t_{1}}^{t}dt^{\prime }Y(t^{\prime })F(t^{\prime })e^{-k(t-t^{\prime })}.$$

If the activated immune response acting on the metastasis has the same effectiveness as on the primary tumor, then *Y*(*t*) = 1. In here, we consider two cases:

1. *Y*(*t*) = constant = *y*_0_ (synchronized immune effect)
2. *Y*(*t*) = constant = *y*_0_, but with a time delay *τ* in the onset of the immune response at the metastatic site.

For case 1, according to the previous analysis, the activated immune response is the leading mechanism for time *t > t*_1_ (i.e., after the direct effect of radiation on the primary tumor), significantly reducing the growth rate compared to the untreated case.

If there is no delay in the abscopal effect, by using the parametrization in Equation 3, for constant *y*_0_ (see Equation 10), one gets the time evolution of the metastasis size (for *t > t*_1_, see the **Supplementary Material**).


(11) $$\frac{V_{M}(t)}{V_{M}(t_{1})}=exp[(\ln\;\frac{V_{\infty }^{p}}{V^{p}(t_{1})}+\lambda -y_{0}c_{0}/k-y_{0}c_{f}/k^{2})(1-e^{-k(t-t_{1})})-\frac{y_{0}c_{1}}{k-c_{2}}e^{-(c2t_{1})}(e^{-c2(t-t_{1})}-e^{-k(t-t_{1})})+(t-t_{1}e^{-k(t-t_{1})})y_{0}c_{f}/k]$$

which depends on two parameters, *y*_0_ and *λ*, since *c*_0_, *c*_1_, *c*_2_, and *c_f_* have been determined by fitting the primary tumor evolution. For *y*_0_ = 1 and *λ* = 1, the immune effect on the metastasis is the same as that on the primary tumor. Although *y*_0_ *<* 1 should suggest a depletion of the immune response, this is not necessarily true since *y*_0_ *>* 1 enhances the late time effect related to the parameter *c_f_* (see Equation 11). Indeed, as shown in ref (22), there is a crucial change of sign of the parameter *c_f_*, for the treatment with 25 Gy, and *y*_0_ *>* 1 increases its role for *t >> t*_1_.

## 3 Results

In this section, we clarify how measurements of the volume changes in the primary tumor and in the metastasis can provide a quantitative indication of the systemic immune response activated by FLASH therapy.

Indeed, the computational method of the previous section permits to evaluate the time evolution of the ratio *V*(*t*)*/V*(*t*_1_) for the primary tumor and metastasis, from time *t*_1_.

The parameters of the primary tumor evolution, for 25 Gy of FLASH therapy (20), have been determined in ref (22). (*V ^p (0^*) = 1): $\ln(V_{\infty }^{p}/V^{p}(0))$ = 2.83, *k^p^* = 0.0421 day^−1^, *c*_0_ = 0.056, *c*_1_ = 0.166, *c*_2_ = 0.158 day^−1^, and *c_f_* = −0.00043 day^−2^. The regrowth starts at *t*_1_ = 5 days after FLASH therapy and *V^p^* (*t*_1_)*/V^p^ (0*) = 0.76.

**Figure 3:**
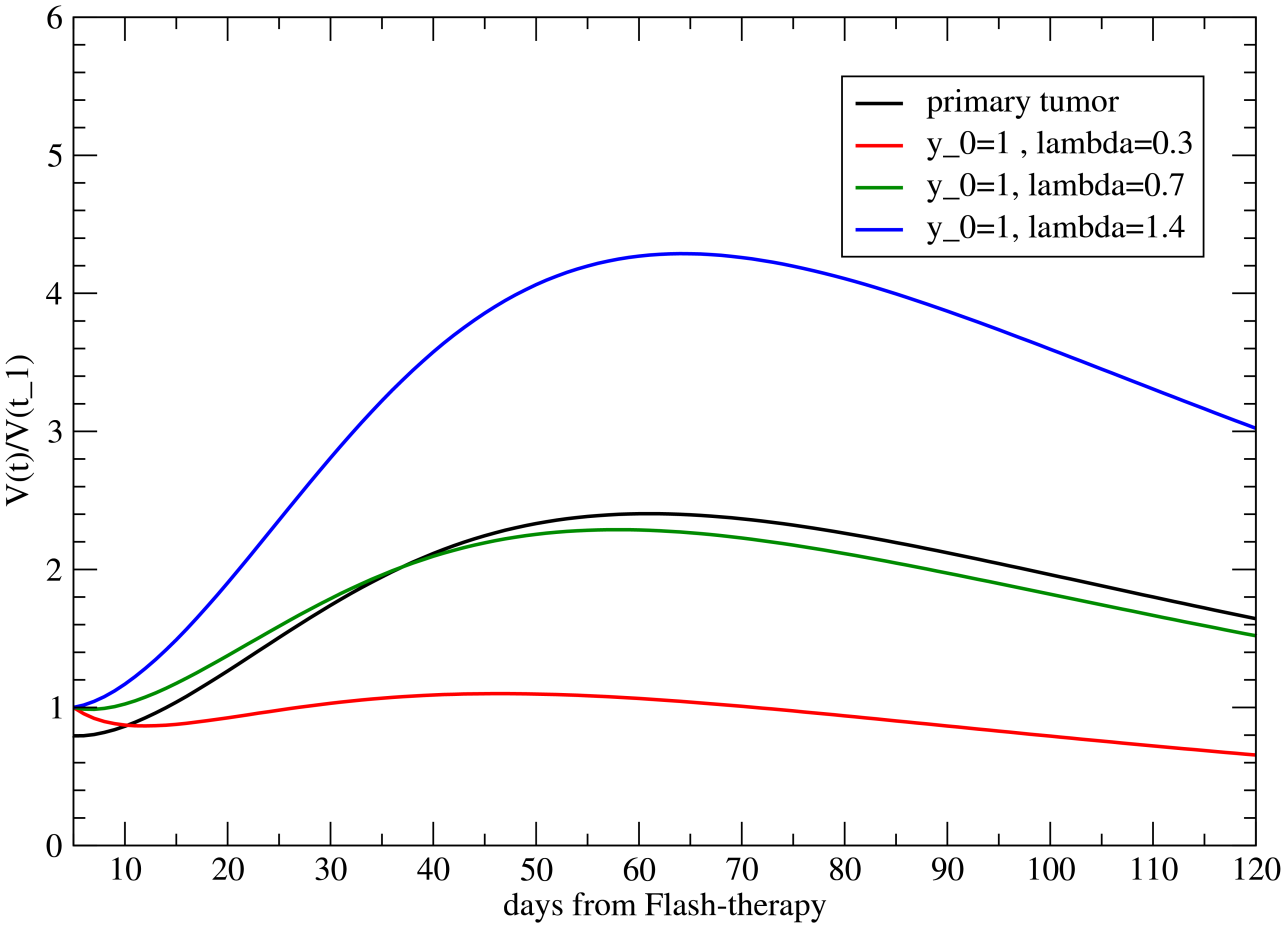
Volume variation with respect to its value at time *t* = *t*_1_ (when the primary tumor starts to regrow) for the metastasis for different values of *λ*, with *y*_0_ = 1.

For no delay, the results for the different values of *λ* for fixed *y*_0_ = 1, based on Equation 11, are depicted in **Figure 3**. Also, a positive *λ* = 1.4 is reported to show its effect. The reference value is *V^M^* (*t*_1_) = 1. The abscopal effect strongly depends on *λ*.

**Figure 4:**
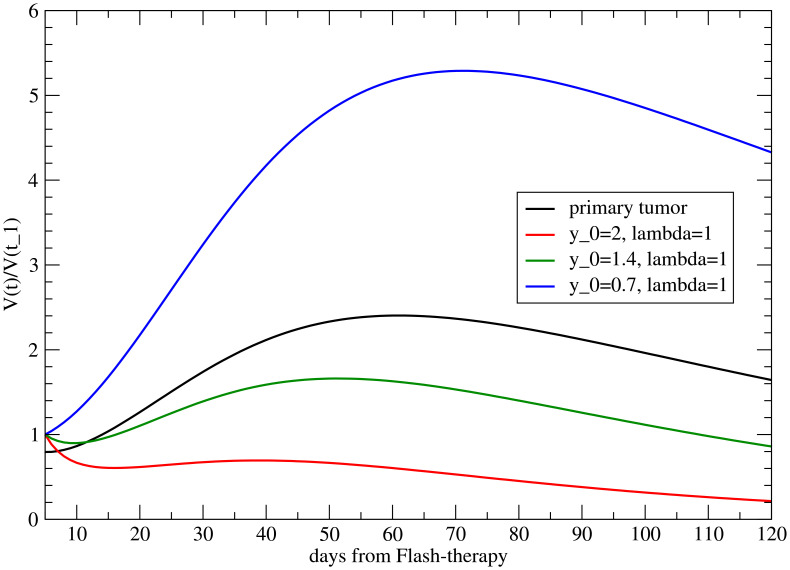
Volume variation with respect to its value at time *t* = *t*_1_ (when the primary tumor starts to regrow) for the metastasis for different *y*_0_, with *λ* = 0.

The analogous results as a function of *y*_0_, for *λ* = 1, are shown in **Figure 4**. As previously discussed, *y*_0_ *>* 1 triggers the abscopal effect, enhancing the late time effect.

The two parameters may be correlated. In fact, although *y*_0_ is time independent, it can depend on the value of the ratio $V_{\infty }^{M}/V^{M}(t_{1})$ , i.e., on *λ*.

The correlation between the two parameters has significant and testable implications. Indeed, the abscopal effect appears to be dependent on tumor size (37). In models with large distal tumors, treatment of the primary tumor produced a strong abscopal response, significantly inhibiting both primary and distant growth. In contrast, in models with small distal tumors, the abscopal effect was minimal, with a little difference observed in combination with irradiation. This difference may reflect variations in the strength of the immune response required for residual inhibition or size-dependent differences in immune cell infiltration.

It is useful to illustrate them with an example.

**Figure 5:**
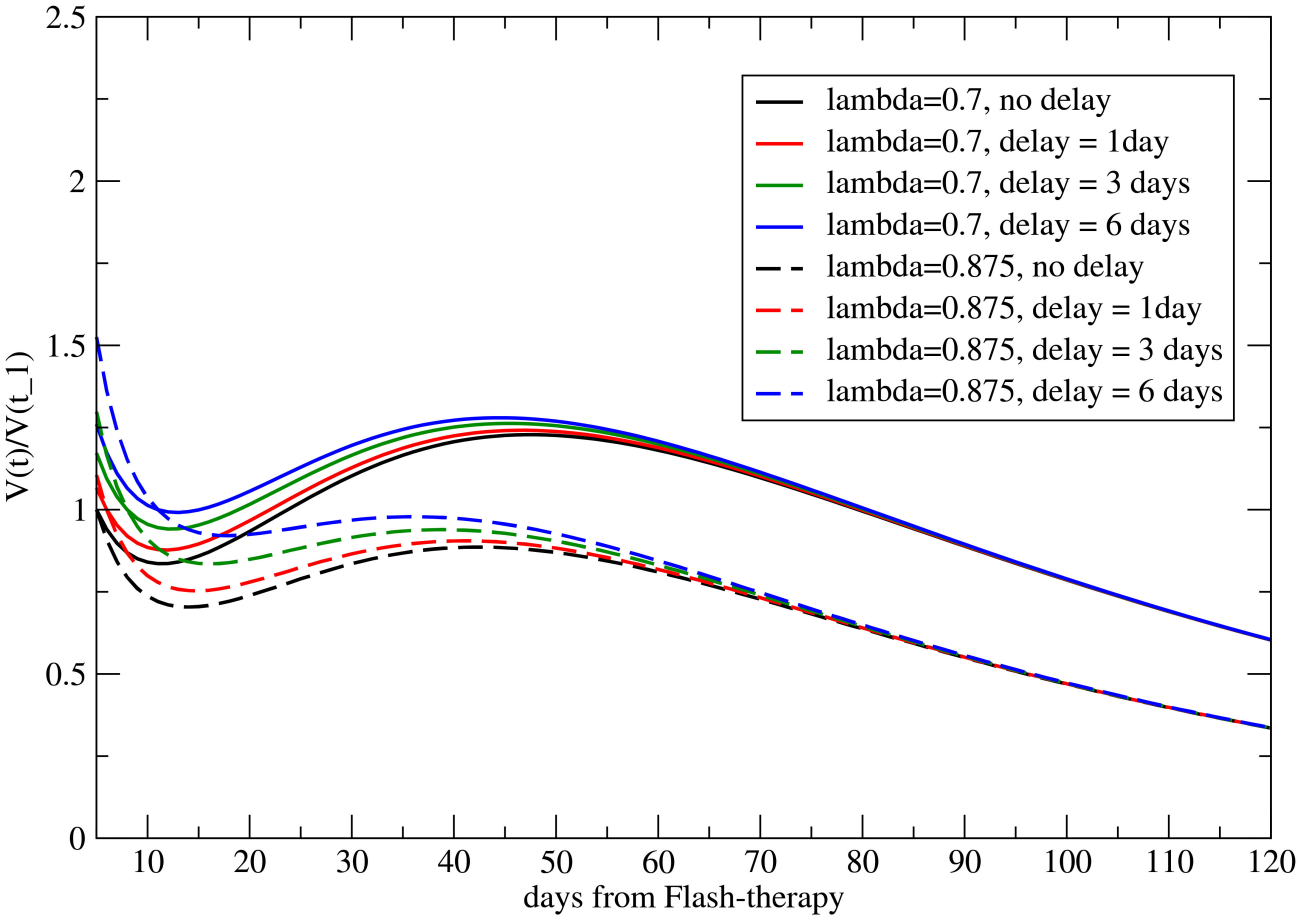
The abscopal effect for different metastatic volumes at *t* = *t*_1_ (i.e., for different values of *λ* and for the correlated parameter *y*_0_ = 2*λ*) when the onset of induced immune response has a delay.

Let us suppose that, for the untreated tumor, the ratio $V_{\infty }^{p}/V^{p}(t_{1})$ and *k* are experimentally determined by fitting the growth curve. If we consider distant metastases located at the same site, it is reasonable to assume that the local carrying capacity, $\;V_{\infty }^{M}$ , is the same for both larger and smaller metastases, i.e., those with larger or smaller volume $V^{M}(t_{1})$ . Under these conditions, *λ* is inversely proportional to $V^{M}(t_{1})$ , and therefore, if the two parameters are not correlated, the abscopal effect is more pronounced for the larger metastasis, as shown in **Figure 5** for *λ* = 0.7 and *λ* = 0.875, corresponding to a volume reduction of 20% at *t* = *t*_1_, for two independent values of *y*_0_. The abscopal effect is stronger for larger metastases (the continuous line is below the dotted line).

**Figure 6:**
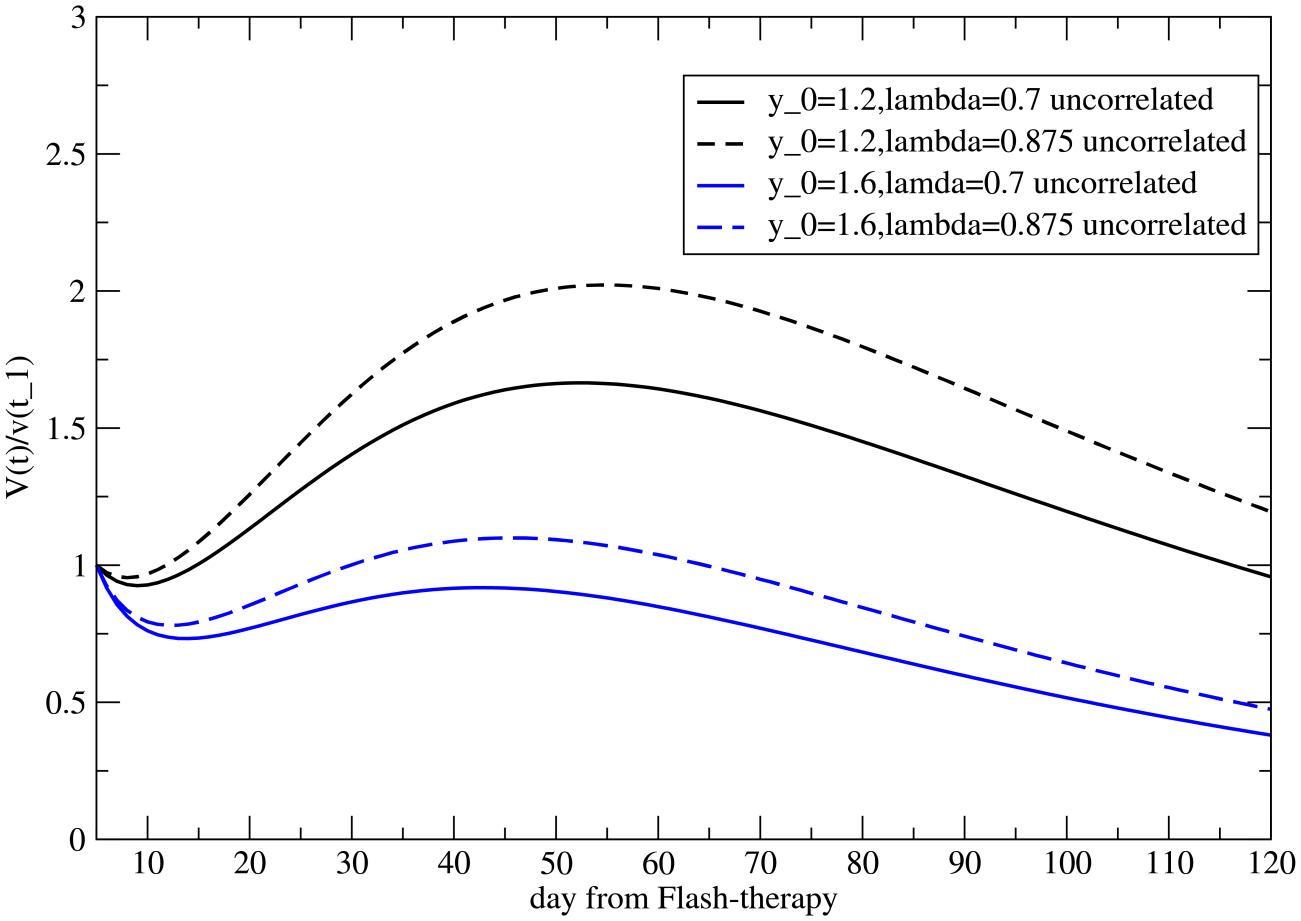
The abscopal effect for different metastatic volumes at the onset of induced immune response (*t > t*_1_), for different values of *λ* and of the uncorrelated parameter *y*_0_. Notice that a smaller value of *λ <* 1 corresponds to a larger *V^M^* (*t*_1_) [see Equation 9].

This result is reversed (the abscopal effect is greater for smaller metastases) if the two parameters are correlated, as shown in **Figure 6**. Therefore, if it is confirmed that in the case of FLASH therapy, the abscopal effect is more pronounced for larger metastases, it would imply that the size of the metastasis is correlated with the effects of the immune system (37).

Notice that **Figures 3**, **4**, and **6** also present the variability of the results, highlighting the sensitivity of the model to modifications in *y*_0_ and *λ*. Regarding the parameters *c*_0_ *,c*_1_,*c*_2_, and *c_f_*, their determination from the data in ref (20). is fairly accurate [see Table 2 in ref (22).], and a variation of one standard deviation results in effects of approximately 10% in the previous figures.

A time delay, *τ*, in the immune response between the immune effect in the primary tumor and the metastatic site implies that *Y*(*t*) = 0 for *t < t*_1_ + *τ* and *Y*(*t*) = *y*_0_ for *t* ≥ *t*_1_ + *τ*. In this scenario, the primary tumor begins to regrow at *t* = *t*_1_, while the growth rate of the metastasis is altered at *t* = *t*_1_ + *τ* due to the delayed onset of the immune response. Therefore, it is more useful to compare the metastasis and primary tumor evolutions after *t* = *t*_1_ using the previous formula (Equation 11) with *t*_1_ → *t*_1_ + *τ* for the metastasis.

**Figure 7:**
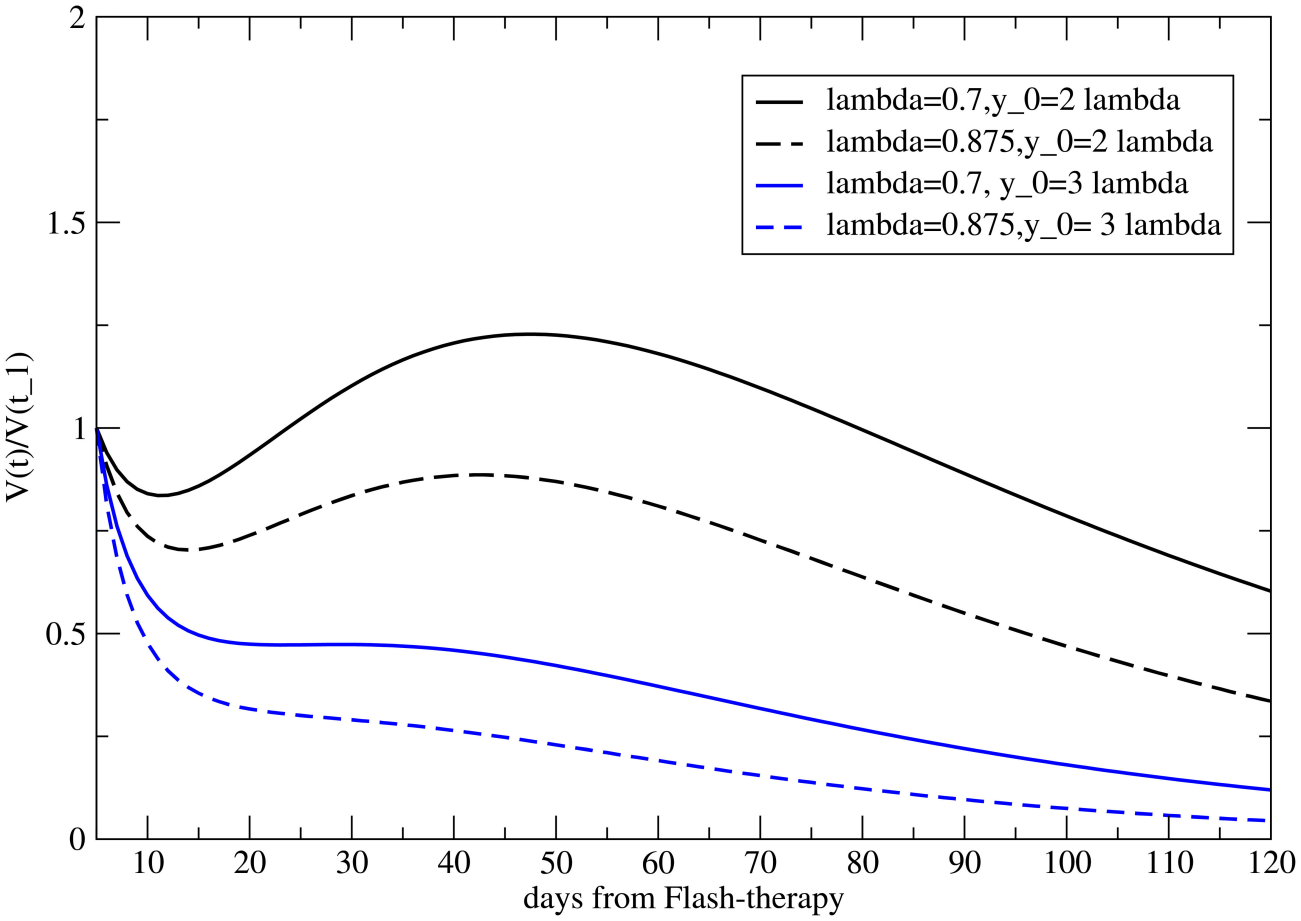
The abscopal effect for different metastatic volumes at the onset of induced immune response (*t > t*_1_), for different values of *λ* and of the correlated parameter *y*_0_. In this case, a smaller metastasis shows a stronger abscopal effect.

The effect of the delay is depicted in **Figure 7** for different values of *λ* and correlated *y*_0_ = 2*λ*. Notice that the conventional value *V_M_* (*t*_1_) = 1 for *τ* = 0 implies that *V_M_* (*t*_1_) *>* 1 for τ≠0 .

## 4 Discussion and conclusions

This work aimed to propose a method to determine to what extent FLASH therapy induces a systemic immune response by monitoring the abscopal effect.

The computational approach for quantitative analysis is based on the following steps, which are informed by experimental data and guided by specific assumptions:

1. Determination of the primary tumor evolution without treatment [as in the experiment reported in ref (20).] to identify the two GL parameters *k* and $V_{\infty }^{p}$ .
2. Estimation of the four parameters, *c*_0_, *c*_1_, *c*_2_, *c*_3_, and *c_f_* by primary tumor evolution after FLASH radiotherapy, as in ref (22).
3. Assumption of a systemic immune effect (38, 39), applying to the metastatic site the specific rate of the regrowth phase of the primary tumor (i.e., *t > t*_1_), while allowing for local variations in the carrying capacity and size of the metastasis (parameter *λ*).
4. Assumption of a constant enhancement or suppression of the immune response (*Y*(*t*) = *y*_0_) at the metastatic site, triggered by FLASH therapy.
5. Inclusion of a time delay in the onset of the immune response between the primary tumor and the metastasis.

Steps (1) and (2) rely on the availability of experimental data. Steps (3) through (5) are model assumptions. The quantitative analysis requires the determination of only two to three additional parameters, which can be easily extracted using standard software tools applied to the metastatic growth data. The correlation between *λ* and *y*_0_ implies a direct connection between the abscopal effect and the size of the metastasis at the onset of the immune response due to FLASH therapy. The delay time gives direct information on the one- or multicompartment pharmacokinetic models.

The proposed computational method is based on the Gompertz growth law (point 1); however, any type of two-parameter sigmoidal curve can be used, such as the logistic curve. The Gompertz law provides a better fit to the available data [see ref (26)]. For completeness, the formulas applicable to the logistic growth curve are reported in **Supplementary Material S3**.

Regarding point 4, the immune response to distant metastases may vary over time rather than remaining constant. However, this possibility can be readily incorporated into the computational model if a single value of *y*_0_ does not adequately fit the metastatic evolution data. This can be achieved by introducing a specific time-dependent form of *Y*(*t*) into Equation 6. For instance, an exponential decrease of the immune response over time is presented in **Supplementary Material S2**.

The computational model relies on a set of parameters that must be specified for the given tumor phenotype in order to quantitatively assess the abscopal effect induced by radiotherapy. Therefore, the experimental procedure requires that the primary tumor is first inoculated in the test animals, followed by injection of the same tumor cells at a distant site to allow metastatic development. FLASH therapy is then applied to the primary tumor, and the evolution of both primary and metastatic tumor volumes is subsequently monitored. Initially, it is essential to fit the primary tumor growth data from two control groups—one untreated and one treated with FLASH therapy alone, without metastasis. This fitting procedure allows for the determination of the parameters *k*, $V_{\infty }^{p}$ , and *c*_0_, *c*_1_, *c*_2_, *c*_3_, and *c_f_* for the tumor phenotype. Subsequent measurements of metastatic volume changes enable the determination of the two parameters *λ, y*_0_ (assuming no delay). However, by measuring both the primary and metastatic tumor volumes at the onset of the regrowth phase, the parameter *λ* becomes fixed—since the carrying capacity for the primary tumor has already been determined—and the fitting procedure then depends only on a single parameter *y*_0_. The delay time *τ* is an additional parameter that can be introduced during data fitting to test whether it plays a significant role. Its relevance can be assessed by evaluating the improvement in the quality of the fit (*χ* ^2^ per degree of freedom) when the delay is included. As discussed, the assumption in point (4) can be relaxed at the cost of introducing additional parameters.

The difference between tumor phenotypes is reflected in the specific values of the parameters in the function *F*(*t*) (regarding the primary tumor) and in the values of the additional parameters *y*_0_ and *λ*, which characterize the induced immunity response on the metastases.

In conclusion, we have proposed a quantitative method to evaluate the abscopal effect associated with FLASH therapy. In our view, this type of treatment should trigger a systemic immune response. Otherwise, it would be difficult to explain why, following the initial sharp reduction in tumor cells, a regrowth phase occurs with a specific rate significantly lower than in the untreated case—and, more importantly, why a subsequent tumor regression takes place long after the treatment has been administered.

The computational method, while open to improvement in several respects, offers a quantitative estimate of deciphering the activated immune response.

## Data Availability

The original contributions presented in the study are included in the article/**Supplementary Material**, further inquiries can be directed to the corresponding author/s.
