# Supplementary material for: Phenomenological insights into the FLASH radiotherapy-induced abscopal effect
Source: Front Oncol. 2025 Sep 17;15:1657392. doi: 10.3389/fonc.2025.1657392 (PMC12486310; doi:10.3389/fonc.2025.1657392)
Supplement: Supplementary file 1 [file DataSheet1.pdf]

Supplementary Material

1 GENERAL FORMULAS

An untreated tumor grows according to the Gompertz law (GL), solution of the equation

$$\frac{1}{V}\frac{dV}{dt} = a - kln(\frac{V(t)}{V(t_0)}) = kln(\frac{V_\infty}{V}), \tag{S1}$$

with two parameters, a, k or k, V_∞, where the carrying capacity, V_∞, is given by $V_\infty/V(t_0) = exp(a/k)$.

The solution is

$$V(t) = V(t_0)e^{(a/k)[1-e^{-k(t-t_0)}]} = V(t_0)e^{ln\frac{V_\infty}{V(t_0)}[1-e^{-k(t-t_0)}]} \tag{S2}$$

Let us consider the role of the therapy $F(t)$, i.e. let us modify the specific growth rate (the second term in eq.(S1)) according to

$$\frac{1}{V}\frac{dV}{dt} = a - kln(\frac{V(t)}{V(t_0)}) - F(t) = kln(\frac{V_\infty}{V}) - F(t) \tag{S3}$$

where the variable $F(t)$ generically refers to the effect of any therapy (radio, immune,chemo) and/or to their combination.

The solution of previous equation is

$$\begin{aligned} V(t) &= V(t_0)e^{(a/k)[1-e^{-k(t-t_0)}]-\int_{t_0}^{t} dt^{'} F(t^{'})e^{-k(t-t^{'})}} \\ &= V(t_0)e^{ln\frac{V_\infty}{V(t_0)}][1-e^{-k(t-t_0)}]-\int_{t_0}^{t} dt^{'} F(t^{'})e^{-k(t-t^{'})}}. \end{aligned} \tag{S4}$$

According to parametrization of $F(t)$ for Flash-therapy,

$$F(t) = c_0 + c_1 * e^{(-c_2 t)} + c_f t, \tag{S5}$$

one gets

$$\begin{aligned} V(t)/V(t_0) = exp[(ln\frac{V_\infty}{V(t_0)} - c_0/k - c_f/k^2)(1 - e^{-k(t-t_0)}) \\ - \frac{c_1}{k-c_2}e^{-c2t_0}(e^{-c_2(t-t_0)} - e^{-k(t-t_0)}) + c_f/k(t - t_0e^{-k(t-t_0)}]. \end{aligned} \tag{S6}$$

The general formula with a delay τ is obtained by the substitution $t_0 \to t_0 + \tau$.

2 TIME DEPENDENT IMMUNE RESPONSE

The immune response to distant metastases may vary over time and not remain constant. This possibility can be easily incorporated into the computational model if a constant value of y_0 fails to fit the metastatic

evolution data. This can be done by introducing a specific form of $Y(t)$ into eq. (6). The choice $Y(t) =$ constant is intended to illustrate the applied methodology. For example, let us assume that

$$Y(t) = y_0 exp(-\gamma t). \tag{S7}$$

The substitution in eq.(6) requires the evaluation of

$$\int_{t_1}^{t} Y(t^{'})F(t^{'})e^{-k(t-t^{'})} \tag{S8}$$

which, by the parametrization in eq.(3), gives

$$\begin{aligned}\int_{t_1}^{t} Y(t^{'})F(t^{'})e^{-k(t-t^{'})} = y_0 exp[(-\gamma t)]\big[\frac{1}{k-\gamma}[1-e^{-(k-\gamma)(t-t_1)}] \\ +\frac{1}{k-c_2-\gamma}exp[-(c_2 t)][1-e^{-(k-c_2-\gamma)(t-t_1)}] \\ +\frac{c_f}{(k-\gamma)^2}[1-e^{-(k-\gamma)(t-t_1)}] \\ -\frac{c_f}{(k-\gamma)}[t-t_1 e^{-(k-\gamma)(t-t_1)}]\big]\end{aligned} \tag{S9}$$

3 LOGISTIC GROWTH AND THERAPY

The logistic growth equation is

$$\frac{dV}{dt} = kV(1-\frac{V}{V_\infty}) \tag{S10}$$

with solution

$$V(t) = \frac{V_0 e^{k(t-t_0)}}{1-(V_0/V_\infty)[1-e^{k(t-t_0)}]} \tag{S11}$$

By including (any) therapy,$F(t)$, one gets the Bernoulli equation

$$\frac{dV}{dt} = kV(1-\frac{V}{V_\infty}) - VF(t). \tag{S12}$$

By the usual substitution $w = 1/V$, one obtains the linear differential equation

$$\frac{dw}{dt} = -[k-F(t)]w - k/V_\infty \tag{S13}$$

with solution

$$w(t) = exp[-M(t,t_0)](w_0 - \frac{k}{V_\infty}\int_{t_0}^{t} exp[M(\hat{t},t_0)]d\hat{t}) \tag{S14}$$

where

$$M(t,t_0) = k(t-t_0) - \int_{t_0}^{t} dt^{'} F(t^{'}). \tag{S15}$$
